# Stabilization of MCL-1 by E3 ligase TRAF4 confers radioresistance

**DOI:** 10.1038/s41419-022-05500-6

**Published:** 2022-12-19

**Authors:** Ming Li, Feng Gao, Xiaoying Li, Yu Gan, Shuangze Han, Xinfang Yu, Haidan Liu, Wei Li

**Affiliations:** 1 Cell Transplantation and Gene Therapy Institute, The Third Xiangya Hospital of Central South University, Changsha, Hunan 410013 People’s Republic of China; 2 Changsha Stomatological Hospital, Changsha, Hunan 410004 People’s Republic of China; 3 School of Stomatology, Hunan University of Chinese Medicine, Changsha, Hunan 410208 People’s Republic of China; 4 Department of Radiology, The Third Xiangya Hospital of Central South University, Changsha, Hunan 410013 People’s Republic of China; 5 Department of Ultrasonography, The Third Xiangya Hospital of Central South University, Changsha, Hunan 410013 People’s Republic of China; 6 Department of Ultrasound, Union Hospital, Tongji Medical College, Huazhong University of Science and Technology, Wuhan, Hubei 430022 People’s Republic of China; 7 Department of Medicine, Baylor College of Medicine, Houston, TX 77030 USA; 8 Department of Cardiovascular Surgery, The Second Xiangya Hospital of Central South University, Changsha, Hunan 410011, People’s Republic of China; 9 Clinical Center for Gene Diagnosis and Therapy, The Second Xiangya Hospital of Central South University, Changsha, Hunan 410011, People’s Republic of China

**Keywords:** Oral cancer, Tumour biomarkers

## Abstract

The E3 ligase TNF receptor-associated factor 4 (TRAF4) is frequently overexpressed and closely related to poor prognosis in human malignancies. However, its effect on carcinogenesis and radiosensitivity in oral squamous cell carcinoma (OSCC) remains unclear. The present study found that TRAF4 was significantly upregulated in primary and relapsed OSCC tumor tissues. Depletion of TRAF4 markedly improved the sensitivity of OSCC cells to irradiation (IR) treatment, showing that tumor cell proliferation, colony formation and xenograft tumor growth were reduced. Mechanistically, IR promoted the interaction between TRAF4 and Akt to induce Akt K63-mediated ubiquitination and activation. TRAF4 knockout inhibited the phosphorylation of Akt and upregulated GSK3β activity, resulting in increased myeloid cell leukemia-1 (MCL-1) S159 phosphorylation, which disrupted the interaction of MCL-1 with Josephin domain containing 1 (JOSD1), and ultimately induced MCL-1 ubiquitination and degradation. Moreover, TRAF4 was positively correlated with MCL-1 in primary and in radiotherapy-treated, relapsed tumor tissues. An MCL-1 inhibitor overcame radioresistance in vitro and in vivo. Altogether, the present findings suggest that TRAF4 confers radioresistance in OSCC by stabilizing MCL-1 through Akt signaling, and that targeting TRAF4 may be a promising therapeutic strategy to overcome radioresistance in OSCC.

## Introduction

Oral squamous cell carcinoma (OSCC) is one of the most common histological subtypes of head and neck cancers, and ranks sixth in terms of the number of cancer-associated mortalities worldwide [1, 2]. Currently, the standard treatment for patients with OSCC is surgery for early-stage cancers, or surgery combined with chemotherapy, radiotherapy or targeted therapy for advanced cancer [3–5]. Although these constantly updated and advanced treatments have improved the quality of life of OSCC patients to a certain extent, the overall survival rate has not improved, and the 5-year survival rate of patients with advanced OSCC remains low (<50%) [6]. Radiotherapy is an important treatment strategy for patients with OSCC. However, radioresistance is often observed, which represents a major impediment for effective treatment [7]. It has been reported that certain genes and molecular pathways are responsible for radiation resistance, while the comprehensive mechanisms of radioresistance in OSCC have not yet been fully elucidated [8].

TNF receptor-associated factor 4 (TRAF4), a unique TRAF family member, was initially identified from human metastatic lymph nodes of breast cancer [9]. Unlike other TRAFs, the biological function of TRAF4 is rarely involved in the development and normal function of the immune system, with the exception of promoting the migration of immune cells [10]. Moreover, TRAF4 is the first member of the TRAF protein family to be amplified and overexpressed in multiple human malignancies, including breast, lung, prostate and colorectal cancer, and OSCC [11–13]. Several lines of evidence revealed that TRAF4 was not only involved in the initiation and progression of tumors, but also in chemoresistance and radioresistance [12, 14–16]. Hao et al. identified that TRAF4 was highly expressed in breast cancer and possessed the function of inhibiting apoptosis and promoting cancer cell proliferation via regulating the ubiquitination of spindle assembly-associated protein Eg5 [17]. A previous study had shown that TRAF4 upregulation was related to the proliferation, invasion, and migration of OSCC cells by inducing the activation of the Wnt/β-catenin signaling pathway [11]. RNA-sequencing, a high-throughput sequencing-based approach, was for the first time used to propose TRAF4 as a novel candidate for radioresistance in non-small cell lung cancer (NSCLC) [14]. However, the crucial role of TRAF4 in radiation resistance and its underlying mechanism in human tumor, including OSCC, remains elusive.

The current study demonstrated that TRAF4 was overexpressed in OSCC tumor tissues and exhibited higher expression in the relapsed tumor specimens after IR treatment. Depletion of TRAF4 impaired the stability of MCL-1 protein and sensitized OSCC cells to radiotherapy in vitro and in vivo. Therefore, targeting TRAF4 could be an attractive strategy to overcome radioresistance in OSCC.

## Materials and methods

### Chemical reagents

The reagents NaCl, sodium dodecylsulfate (SDS), Tris and dimethyl sulfoxide (DMSO) were purchased from Sigma-Aldrich (St. Louis, MO, USA). Cell culture media DMEM medium and supplements FBS and penicillin-streptomycin were from Invitrogen (Grand Island, NY). The compounds MG132, cycloheximide (CHX), SB216763 (#S1075), and S63845 were obtained from Selleck Chemicals (Houston, TX).

### Cell lines and cell culture

CAL27, SCC25 and 293T cells were purchased from American Type Culture Collection (ATCC, Manassas, VA) and cultured according to the manufacturer’s instructions in DMEM medium supplemented with 10% Fetal Bovine Serum (FBS) and 1% penicillin-streptomycin (all from Invitrogen; Thermo Fisher Scientific, Inc.). All cells were maintained at 37 ˚C in a humidified incubator with 5% CO_2_, assessed for mycoplasma, and tested cytogenetically every 2 months. The radioresistant cell lines CAL27R and SCC25R were established in our laboratory, as previously described [18]. Briefly, CAL27 and SCC25 cells were serially exposed to an increasing dose of IR, from 2 Gy/day, to a final dose of 80 Gy, for ~6 months.

### Cell viability assay

MTS assay was used to analyze the viability of OSCC cells. Briefly, OSCC cells (2 × 10^3^ cells/well) were seeded into a 96-well plate and treated with IR (2 Gy) or S63845 inhibitor (2 μM). After incubation for 48 h at a 37 ˚C incubator, MTS regent (#G3581, Promega Corporation) was added to each well and cultured for another 1 h. Cell viability was then measured following the standard protocol.

### Plate colony formation assay

OSCC cells were treated with IR (2 Gy) or S63845 (2 μM), and followed by incubation cells in a 6-well plate (500 cells/well) at 37 ˚C in the presence of 5% CO_2_ for 2 weeks. When visible colonies appeared on the plate, 4% paraformaldehyde was added to fix the colonies for 20 min at 37 ˚C, and 0.5% crystal violet was then used to stain the colonies for 5 min at 37 ˚C. The numbers of colonies were counted under a microscope.

### Anchorage-independent cell proliferation assay

OSCC cells (8 × 10^3^ cells/well) were treated with IR (2 Gy) or S63845 (2 μM), and suspended in 1 ml of 0.3% agar with Eagle’s medium containing 10% FBS. The cell suspension was added to a 6-well plate containing a 0.6% agar base. Plates were maintained in a 5% CO_2_ incubator at 37 ˚C for 2 weeks before the colony number was counted microscopically.

### Immunoblotting (IB) and immunoprecipitation (IP) assay

Whole-cell extraction (WCE) was performed utilizing pre-cooled RIPA buffer (#89900; Thermo Fisher Scientific, Inc.) with protease inhibitors. A BCA protein assay kit (#23225, Thermo Fisher Scientific, Inc.) was used to determine the protein concentration according to the instructions of the manufacturer. For the co-immunoprecipitation (Co-IP) assay, the cell lysates were prepared with the IP buffer (#87787, Thermo Fisher Scientific, Inc.), an equal quantity of cell lysate was incubated with the corresponding primary antibody and Protein A/G agarose beads. The agarose beads were washed with ice-cold PBS and resuspended in 40 µl 1X SDS-PAGE loading buffer to collect the supernatant. The extracted proteins were separated by SDS-PAGE electrophoresis and then transferred to polyvinylidene difluoride membranes. After blocking with 5% non-fat milk for 1 h at 37 ˚C, the membranes were incubated with the primary antibody overnight at 4 ˚C, followed by the appropriate horseradish peroxidase-conjugated secondary antibody for 1 h at room temperature. Protein bands were visualized using the enhanced chemiluminescence reagents (#32132, Thermo Fisher Scientific, Inc.). The primary and secondary antibodies against γ-H2A histone family member X (H2AX) (#9718, 1:4000), cleaved-caspase 3 (#9661, 1:1000), cleaved-poly (ADP-ribose) polymerase (PARP) (#5625, 1:1000), MCL-1 (#5453, 1:1000), phosphorylated (p)-MCL-1-Ser159 (#ab111574, 1:1000), ubiquitin (#3936, 1:1000), Akt (#4691, IB: 1:1000; IP: 1:200), p-Akt-Ser473 (#4060, 1:1000), GSK3β (#12456, 1:1000), p-GSK3β-Ser9 (#5558, 1:1000), MCL-1(#5453, IB: 1:1000, IP: 1:200), USP13 (#12577, 1:1000), USP9X (#14898, 1:1000), Ku70 (#4588, 1:1000), β-actin (#3700, 1:10,000), rabbit horseradish peroxidase (HRP)-conjugated IgG (#7074, 1:10000), and mouse HRP-conjugated IgG (#7076, IB, 1:10,000) were purchased from Cell Signaling Technology, Inc. (Beverly, MA). The antibodies against TRAF4 (#MABC985, IB: 1:4000, IP: 1:200) and Flag tag (#F3165, 1:10000) were obtained from Sigma-Aldrich (St. Louis, MO). The antibodies against DUB3 (#ab112436, 1:1000), JOSD1 (#ab118221, IB: 1:1000, IP: 1:200), HA tag (#ab18181, 1:5000) and His tag (#ab18184, 1:5000) were purchased from Abcam (Cambridge, UK).

### Plasmid construction

Flag-TRAF4 (#RC200345) and Flag-MCL1 (#RC200521) were obtained from OriGene Technologies, Inc. His-Ub (#31815), HA-Akt (#73408), HA-Ub-K48 (#17605), and Flag-JOSD1 (#22547) were obtained from Addgene (Watertown, MA, USA). Flag-TRAF4 (C18A), HA-Ub-K48R, His-Ub (K6, K11, K27, K29, K33, K48, and K63), HA-K8/K14R, Flag-MCL1 (S159D), and Flag-MCL1 (S159A) mutant were obtained using the Q5 Site-Directed Mutagenesis Kit (cat. #E0554S; New England BioLabs, Inc.) according to the manufacturer’s instructions. All mutant constructs were generated using mutagenic PCR and verified by Sanger DNA sequencing.

### Transient transfection and construction of knockout stable cell lines

Lipofectamine® 2000 (Invitrogen, Thermo Fisher Scientific, Inc.) was used for transient transfection following the manufacturer’s instructions. Small hairpin (sh)GFP (#110318, Addgene, Inc.) was used as sh control (Ctrl). GSK3β stable knockdown cells were generated using shRNA (5’-CATGAAAGTTAGCAGAGAT-3’). The shGSK3β lentivirus plasmid, psPAX2, and pMD2.G were co-transfected into 293 T cells for 48 h, and the samples were then centrifuged and filtered to collect the virus-containing supernatant. The supernatant of virus-infected OSCC cells was incubated with 4 µg/ml polybrene overnight and the cells were selected with 1 µg/ml puromycin for 2 weeks. OSCC cells were seeded in 6-well plates and transfected with MCL1 small interfering (si)RNA (sc-35877, Santa Cruz Biotechnology, Inc.) and Ctrl siRNA (sc-37007, Santa Cruz Biotechnology, Inc.) using HiPerFect transfection reagent (Cat. 301705, Qiagen, Inc.) as previously described [19]. After 48 h, western blotting was performed to confirm that MCL-1 was knocked down.

The CRISPR-Cas9 technique was used to generate gene knockout stable cell lines as previously described [20]. The cloning oligos were subcloned into skeleton vector lentiCRISPR v2 to establish TRAF4 knockout constructs (sgTRAF4#1: oligo 1, 5′-AGCCACAAAACTCGCACTTG-3′ and oligo 2, 5′-CAAGTGCGAGTTTTGTGGCT-3′; and sgTRAF4#2: oligo 1, 5′-CTCTGCCCATTCAAAGACTC-3′ and oligo 2, 5′-GAGTCTTTGAATGGGCAGAG-3′). The sgTRAF4 plasmids were transfected to generate TRAF4 knockout stable cells, which were selected in 1 μg/ml puromycin-containing medium for 3 weeks.

### Clinical tissue sample collections

OSCC patients were diagnosed and classified by the Department of Pathology at Xiangya Hospital according to the World Health Organization (WHO) guidelines. All surgical specimens were collected according to protocols approved by the Institutional Review Board. Individuals included 81 cases of primary oral squamous cell carcinoma with matched adjacent normal tissue, and 20 cases of relapse after receiving radical radiotherapy (70 Gy). Written informed consent was obtained from all patients.

### Ubiquitination analysis

The Ubiquitination analysis was performed as described previously [21]. Briefly, Cells co-transfected with the indicated plasmids were harvested. The cells were lysed with RIPA buffer (20 mM NAP, pH7.4, 150 mM NaCl, 1% Triton, 0.5% Sodium-deoxycholate, and 1% SDS) supplemented with protease inhibitors and 10 mM N-ethylmaleimide (NEM) for IP-mediated ubiquitination analysis; and with Ni-NTA lysis buffer (6 M guanidine-HCl, 0.1 M Na_2_HPO_4_/NaH_2_PO_4_, 0.01 M Tris/HCl, pH 8.0, 5 mM imidazole, and 10 Mm β-mercaptoethanol) supplemented with 10 mM NEM and protease inhibitors for Ni-NTA pull-down assay. Protein ubiquitination levels were determined by IB analysis

### Immunohistochemical (IHC) staining

The tumor tissues from xenograft tumors or OSCC patients were subjected to IHC staining as described previously [22]. Briefly, the tissue slides were dewaxed in xylene and rehydrated using different ethanol concentrations. Followed by Antigen retrieval in sodium citrate buffer (10 mM, pH6.0), the slides were incubated with 3% H_2_O_2_ in methanol to block endogenous peroxidase. After blocking with 50% goat serum albumin, slides were incubated with Ki67 (#ab16667, Abcam, 1:250), TRAF4 (#MABC985, Sigma-Aldrich, 1:300), MCL-1 (#ab32087, Abcam, 1:100) primary antibodies overnight, and then hybridized with the secondary antibody from Vector Laboratories (Burlingame, CA) (anti-rabbit 1:200). The tissues were counterstained with hematoxylin. Slides were visualized and analyzed by two senior pathologists with a microscope and Image-Pro Plus software (version 6.2) program (Media Cybernetics), respectively. The stained tissues were quantified by a comprehensive score, which depended on the number of positive cells and the intensity of the dye color. The percentage of positive cells was divided into four categories: 0, no positive cells; 1, ≤10% positive cells; 2, 10–50% positive cells; 3, >50% positive cells. The intensity was graded as 0, no staining; 1, weak staining; 2, moderate staining; 3, intense staining. The comprehensive score of TRAF4 and MCL-1 expression: ≤2 indicates low expression level; >2 indicates high expression level.

### 5-ethynyl-2′-deoxyuridine (EdU) incorporation assay

EdU assay was performed to measure OSCC cell proliferation. Cells were seeded in the 96-well plate at 1 × 10^4^ cells/well and treated with or without IR (2 Gy). The cells were incubated with 10 μM of EdU (C10339, Thermo Fisher) at 37 ˚C for 2 h, then fixed in 4% paraformaldehyde and permeabilized in 0.5% Triton X-100 for 30 min. After blocking in 5% BSA for 1 h, 100 μl of staining solution was added to each well and incubated in the dark for 1 h. Next, nuclei were counterstained with DAPI (P36935, Thermo Fisher Scientific). Images were analyzed using the confocal fluorescence microscope system (Nikon C1si; NIKON Instruments Co.).

### Xenograft mouse model

All animal protocols were approved by the Institutional Animal Care and Use Committee (IACUC) of Central South University (Changsha, China). For the tumor xenograft model of OSCC, CAL27R cells (2 × 10^6^) in 200 μl DMEM were harvested and were subcutaneously injected into the right flank of 6-week-old athymic nude mice (*n* = 5). Tumor growth was quantified with a digital caliper every 3 days, and tumor volume was calculated as length × width^2^ × 0.5. When the tumor volume reached 100 mm^3^, the mice were randomly allocated to different groups, and IR treatment (2 Gy) was started. IR was performed with X-RAD 320 (Precision X-ray, Inc.) at a dosage of 2 Gy twice per week. For MCL-1 inhibitor S63485 treatment, the tumor-bearing mice were randomly divided into four groups (*n* = 5): 1, vehicle control (0.5% dimethyl sulfoxide in Corn oil, 100 µL/every 2 days, i.p.); 2, S63845 (5 mg/kg/ in 100 µL Corn oil every 2 days, i.p.); 3, local ionizing radiation (2 Gy/ twice per week); 4, S63845 (5 mg/kg/ in 100 µL Corn oil every 2 days, i.p.) + local ionizing radiation (2 Gy/ twice per week). Finally, the mice were euthanized, and the tumor tissues were collected for weight recording, IHC staining and western blotting analysis.

### Statistical analysis

All experiments in this study were performed in triplicate. Data were presented as means ± SEM of three independent experiments. SPSS (version16.0 for Windows, SPSS Inc, Chicago, IL, USA) and GraphPad Prism 5 (GraphPad 5.0, San Diego, CA, USA) were used for data analyses. The Student’s *t* test or ANOVA assessed the difference between means. The χ ^2^ test or Fisher exact test was used to evaluate clinicopathologic significance in clinical samples for categorical data. Mann–Whitney *U* test was used when the data did not fit a normal distribution. The Pearson rank correlation was used for correlation tests. The expression level difference between adjacent and tumor was evaluated by Wilcoxon matched-pairs signed-rank test. For all statistical analysis, *p* < 0.05 was considered statistically significant.

## Results

### TRAF4 is required for maintaining tumorigenic properties and IR resistance of OSCC cells

**Fig. 1:**
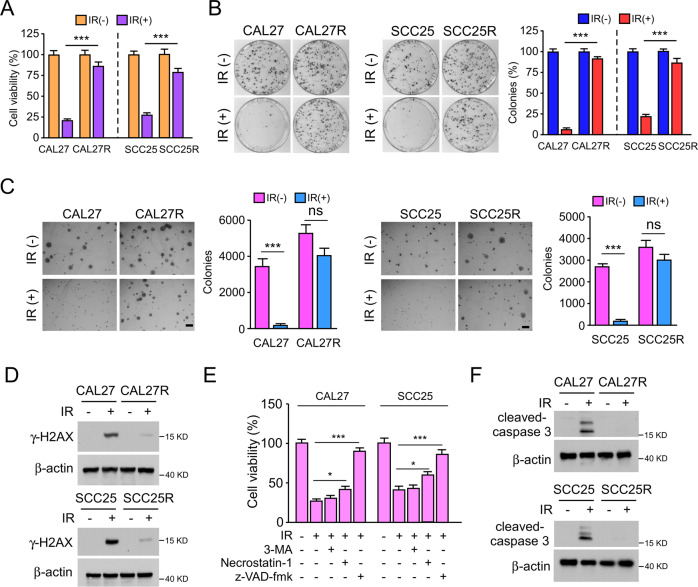
Irradiation induces apoptosis in OSCC cells. **A** MTS assay was used to determine the cell viability of CAL27/CAL27R and SCC25/SCC25R cells treated with/without IR (2 Gy) for 72 h. **B** Plate colony formation assay was performed to analyze the colony formation of CAL27/CAL27R and SCC25/SCC25R cells treated with/without IR (2 Gy) for 72 h. **C** Soft agar assay was employed to assess the anchorage-independent cell proliferation of CAL27/CAL27R and SCC25/SCC25R cells treated with/without IR (2 Gy) for 72 h. **D** Immunoblotting for γ-H2AX expression in CAL27/CAL27R and SCC25/SCC25R cells treated with/without IR (2 Gy) for 72 h. **E** z-VAD-fmk rescued IR-reduced cell viability. CAL27 and SCC25 cells were pretreated with z-VAD-fmk, Necrostatin-1, or 3-MA for 4 h, followed by IR (2 Gy) treatment for 72 h. **F** CAL27/CAL27R and SCC25/SCC25R cells were treated with/without IR (2 Gy) for 72 h. The cell lysates were prepared with the IP buffer. Cleaved-caspase 3 expression was determined by immunoblotting. All data are means ± s.e.m. *p < 0.05, ***p < 0.001, ns not statistically significant. a significant difference between groups as indicated.

As previously reported, resistance to radiotherapy is a primary factor for treatment failure in OSCC [23]. To better understand this observation, radioresistant OSCC cell lines CAL27R and SCC25R were established using the parental CAL27 and SCC25 cells, respectively. The MTS data showed that the viability of CAL27R and SCC25R cells was not significantly decreased after IR treatment (2 Gy) (Fig. 1A). The plate colony formation potential and anchorage-independent cell proliferation in soft agar of both CAL27R and SCC25R cells were not affected by IR treatment, while those of the parental CAL27 and SCC25 cells were impaired (Fig. 1B, C). Furthermore, the protein levels of the DNA-damage marker γ-H2AX in irradiated OSCC cell lines were examined by western blotting. The results indicated that only weak signals were detected in CAL27R and SCC25R cells after IR treatment for 72 h (Fig. 1D). Next, to investigate how IR promotes OSCC cell death, the three most common cell death inhibitors, including the autophagy inhibitor 3-MA, the necroptosis inhibitor necrostatin-1, and the apoptosis inhibitor z-VAD-fmk, were used to pretreat CAL27 and SCC25 cells. As shown in Fig. 1E, z-VAD-fmk significantly restored cell viability after IR-treatment, indicating that activation of intrinsic apoptosis is the main pathway for IR-induced OSCC cell death. Consistently, the IB data revealed that the protein levels of cleaved-caspase 3 were increased in the presence of IR in both parental CAL27 and SCC25 cells, but not in of CAL27R or SCC25R cells (Fig. 1F).

**Fig. 2:**
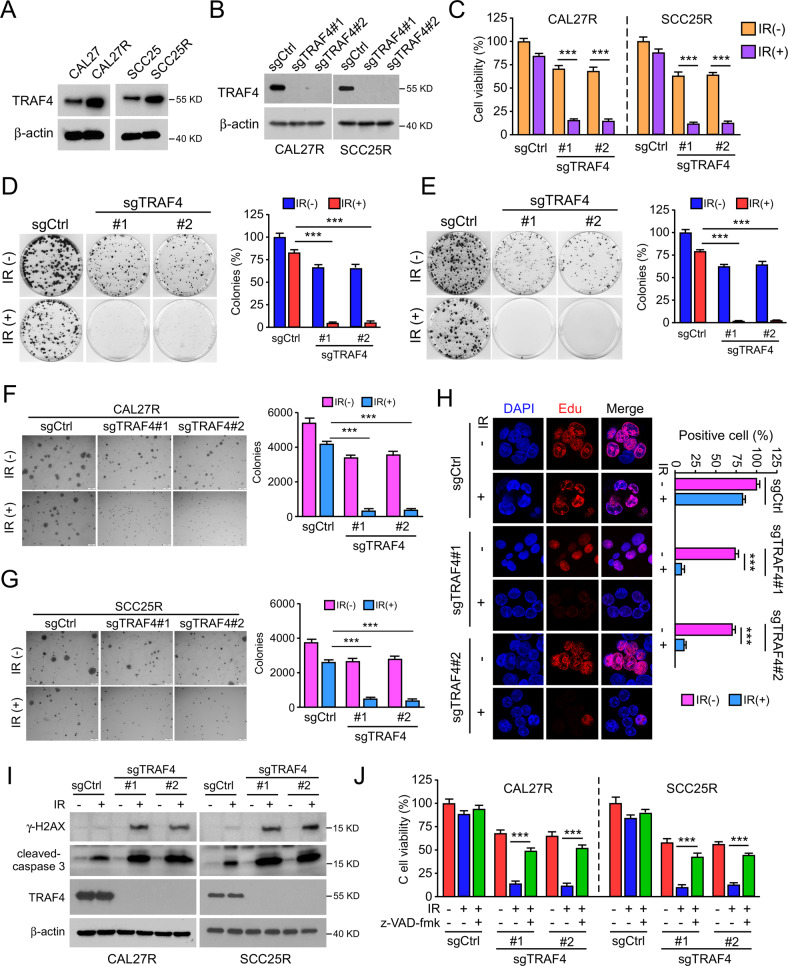
TRAF4 is required for maintaining tumor cells radioresistance. **A** Immunoblotting for TRAF4 expression in CAL27/CAL27R and SCC25/SCC25R cells. **B** Immunoblotting for TRAF4 expression in TRAF4 knockout CAL27R and SCC25R cells. **C** MTS assay was used to determine the cell viability of TRAF4 knockout in CAL27R and SCC25R cells treated with/without IR (2 Gy) for 72 h. **D**, **E** Plate colony formation assay was performed to analyze the colony formation of TRAF4 knockout in CAL27R (**D**) and SCC25R (**E**) cells treated with/without IR (2 Gy) for 72 h. **F**, **G** Soft agar assay were employed to assess the anchorage-independent cell proliferation of TRAF4 knockout in CAL27R (**F**) and SCC25R (**G**) cells treated with/without IR (2 Gy) for 72 h. **H** The representative image of the Edu incorporation assay for TRAF4 knockout in CAL27R cells treated with/without IR (2 Gy) for 72 h. **I** TRAF4-null CAL27R and SCC25R cells were treated with/without IR (2 Gy) for 72 h. WCE was prepared with the RIPA buffer. γ-H2AX and cleaved-caspase 3 expressions were determined by immunoblotting. **J** Pretreated with z-VAD-fmk inhibitor for 4 h, MTS assay was used to determine the cell viability of TRAF4 knockout in CAL27R and SCC25R cells treated with/without IR (2 Gy) for 72 h. All data are means ± s.e.m. ***p < 0.001, a significant difference between groups as indicated.

Notably, the western blot data showed that the E3 ligase TRAF4 was upregulated in radioresistant CAL27R and SCC25R cells compared with the levels found in parental cells (Fig. 2A). To determine whether TRAF4 was required for radioresistance in OSCC cells, TRAF4 stable knockout CAL27R and SCC25R cells were constructed (Fig. 2B). Depletion of TRAF4 markedly suppressed the proliferation capacity of radioresistant cells, as evidenced by decreased cell viability (Fig. 2C), plate colony formation (Fig. 2D, E), anchorage-independent cell proliferation (Fig. 2F, G) and Edu incorporation efficacy (Fig. 2H). IR restored the effect of inducing DNA damage and activating apoptosis in radioresistant OSCC cells, as the protein levels of γ-H2AX and cleaved-caspase 3 were increased after TARF4 depletion (Fig. 2I). Additionally, pretreatment with z-VAD-fmk significantly rescued IR-reduced cell viability in TRAF4-null CAL27R and SCC25R cells (Fig. 2J). These results suggest that TRAF4 is required for the radioresistance of OSCC cells.

### TRAF4 deficiency enhances IR-induced MCL-1 destruction

**Fig. 3:**
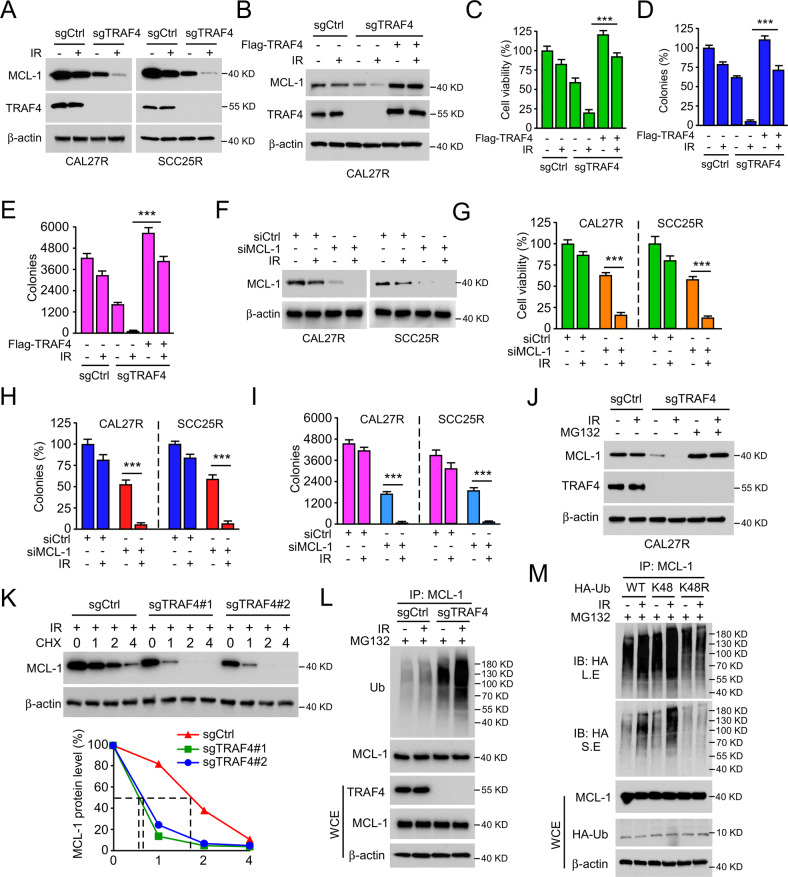
Depletion of TRAF4 reduces IR-induced downregulation of MCL-1. **A** Immunoblotting for MCL-1 expression in TRAF4 knockout CAL27R and SCC25R cells treated with/without IR (2 Gy) for 72 h. **B** TRAF4 was transiently transfected into TRAF4 knockout CAL27R cells for 24 h, then treated with IR (2 Gy) and cultured for 72 h. Immunoblotting was used to detect MCL-1 and TRAF4 expression. **C**–**E** Ectopic expression TRAF4 compromised IR-induced tumor cell growth inhibition. TRAF4 knockout CAL27R cells were transfected with Flag-TRAF4 for 24 h, then treated with IR (2 Gy) and cultured for 72 h. MTS assay was used to determine the cell viability (**C**), plate colony formation assay to analyze the colony formation (**D**) and soft agar assay to assess the anchorage-independent cell growth (**E**). **F** CAL27R and SCC25R were transfected with siMCL1 for 24 h, followed by IR (2 Gy) treated and cultured for 72 h. Immunoblotting was performed to detect MCL-1 expression. **G**–**I** Silencing of MCL-1 inhibited the growth of tumor cells. CAL27R and SCC25R cells were transfected with siMCL1 for 24 h, followed by IR (2 Gy) treated and cultured for 72 h. MTS assay was performed to determine the cell viability (**G**), plate colony formation assay to analyze the colony formation (**H**) and soft agar assay to assess the anchorage-independent cell growth (**I**). **J** Immunoblotting for MCL-1 expression in CAL27R cells treated with IR (2 Gy) for 72 h, followed by incubation with MG132 (25 µM) for 6 h. **K** Immunoblotting for MCL-1 expression in CAL27R cells treated with IR (2 Gy) and incubated with CHX for different time points. **L** TRAF4 knockout CAL27R cells were treated with MG132 (25 μM) for 6 h, then with IR (2 Gy) for 1 h. Cell extracts were subjected to immunoprecipitation (IP) assay to analyze MCL-1 ubiquitination level. **M** 293T cells were transfected with HA-Ub, HA-Ub-K48, and HA-Ub-K48R for 48 h, followed by IR (2 Gy) treated for 20 min. IP assay was performed to detect MCL-1 ubiquitination level. L.E long exposure, S.E short exposure. All data are means ± s.e.m. ***p < 0.001, a significant difference between groups as indicated.

As aforementioned, depletion of TRAF4 sensitized radioresistant cells to radiation, which was largely attributable to the activation of the apoptotic pathway. Next, it was found that the expression of the antiapoptotic protein MCL-1 was reduced in TRAF4-deficient CAL27R and SCC25R cells exposed to IR (Fig. 3A), and this effect was compromised by ectopic overexpression of TRAF4 (Fig. 3B). Reintroduction of TRAF4 in TRAF4-null CAL27R and SCC25R cells markedly rescued IR-reduced cell viability (Fig. 3C) and colony formation (Fig. 3D, E). We speculated that MCL-1 may be a candidate protein regulated by TRAF4 for radioresistance. Following silencing of MCL-1 (Fig. 3F), cell viability (Fig. 3G) and colony formation (Fig. 3H, I) were significantly decreased in the presence of IR in CAL27R and SCC25R cells, which was similar to the effect of TRAF4 knockout. Since ubiquitination is required for MCL-1 degradation [24], TRAF4-null CAL27R cells were treated with the proteasome inhibitor MG132, and it was observed that incubation with MG132 restored the protein expression of MCL-1 after IR treatment (Fig. 3J). Cycloheximide (CHX) protein tracking experiments were carried out to analyze the effect of TRAF4 on MCL-1 stability in CAL27R cells. The results showed that depletion of TRAF4 shortened the half-life of MCL-1 prominently (Fig. 3K). Notably, the ubiquitination assay indicated that the ubiquitination levels of MCL-1 were increased following TRAF4 knockout and were further enhanced upon IR treatment in CAL27R cells (Fig. 3L). As previously stated, K48 is the most common ubiquitin linkage and is usually associated with protein degradation [25]. To determine whether K48 linkage was responsible for IR-induced MCL-1 degradation, ubiquitin mutants K48R (lysine to arginine) and K48 (K48 only) were constructed. The results showed that the K48R, but not the K48 mutant, markedly reduced IR-induced MCL-1 ubiquitination in CAL27R cells (Fig. 3M), indicating that IR-induced MCL-1 polyubiquitination was K48-linked. These data indicate that TRAF4 is required to maintain MCL-1 stability, and TRAF4 knockout enhances IR-induced MCL-1 ubiquitination and degradation.

### Akt/GSK3β signaling is essential for TRAF4 knockout-induced MCL-1 degradation

**Fig. 4:**
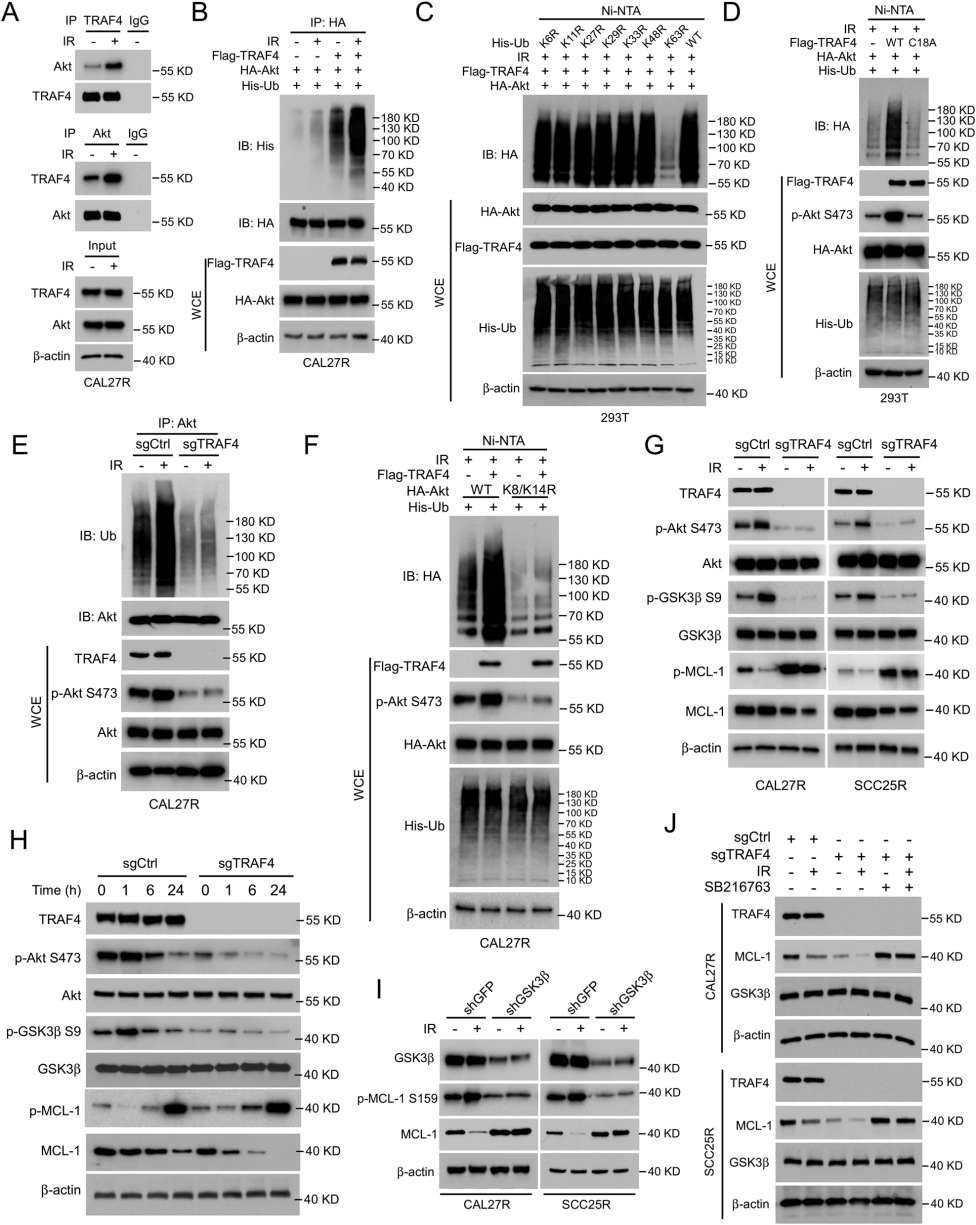
Akt/GSK3β signal pathway is essential for TRAF4 knockout-mediated MCL-1 degradation. **A** Immunoblotting for IP-mediated TRAF4 and Akt expression in CAL27R cells treated with/without IR (2 Gy) for 20 min. **B** CAL27R cells were co-transfected with Flag-TRAF4 and HA-Akt for 48 h, followed by IR (2 Gy) treated for 20 min. Immunoblotting was performed to analyze IP-mediated Akt ubiquitination. **C** 293T cells were co-transfected with Flag-TRAF4, HA-Akt and His-ubiquitin mutant (K6R, K11R, K27R, K29R, K33R, K48R, K63R, WT) for 48 h, followed by IR (2 Gy) treated for 20 min. Ni-NTA pull-down assay was performed to detect Akt ubiquitination. **D** 293T cells were co-transfected with Flag-TRAF4 WT or Flag-TRAF4 C18A mutant, HA-Akt and His-Ub for 48 h, followed by IR (2 Gy) treated for 20 min. Ni-NTA pull-down assay was performed to detect Akt ubiquitination. **E** Immunoblotting for IP-mediated Akt ubiquitination in TRAF4 knockout CAL27R cells treated with or without IR (2 Gy) for 20 min. **F** CAL27R cells were co-transfected with Flag-TRAF4, HA-Akt WT, or HA-Akt K8/K14R mutant and His-Ub for 48 h, followed by IR (2 Gy) treated for 20 min. Ni-NTA pull-down assay was performed to detect Akt ubiquitination. **G** TRAF4 knockout CAL27R and SCC25R cells were treated with/without IR (2 Gy) for 20 min, and WCE was subjected to immunoblotting analysis. **H** TRAF4 knockout CAL27R cells were treated with IR (2 Gy) for different time points, and WCE was subjected to immunoblotting analysis. **I** GSK3β depleted CAL27R and SCC25R cells were treated with IR (2 Gy) for 20 min, and WCE was subjected to immunoblotting analysis. **J** TRAF4 knockout CAL27R and SCC25R cells were treated with SB216763 inhibitor for 24 h, followed by IR (2 Gy) treated for 20 min. WCE was subjected to immunoblotting analysis.

Our group previously found that TRAF4 was required for EGF-induced Akt activation [26]. To determine the potential signal alterations responsible for IR-induced MCL-1 degradation, the present study first examined the changes in Akt signaling in radioresistant OSCC cells. The Co-IP results revealed that IR significantly enhanced the interaction between TRAF4 and Akt in CAL27R cells (Fig. 4A). Moreover, TRAF4-induced Akt ubiquitination was enhanced with IR treatment (Fig. 4B). In total, 7 ubiquitin Lys residues (K6, K11, K27, K29, K33, K48, and K63) were demonstrated to be involved in the ubiquitination of substrate proteins to determine their fate [27]. To investigate which ubiquitination chain linkage participated in TRAF4-mediated Akt ubiquitination after IR treatment, the current study constructed a single-site ubiquitin mutant of those 7 Lys residues. As shown in Fig. 4C, only the K63R mutation significantly reduced the ubiquitination of Akt, indicating that TRAF4-induced Akt ubiquitination after IR treatment is K63-linked.

A previous study on the activation of Akt demonstrated that Lys63-linked ubiquitination was necessary for Akt activity [28]. Thus, we hypothesized that TRAF4 exerted a critical role in regulating Akt activity in the presence of IR. The Ni-nitrilotriacetic acid (NTA) pull-down assay indicated that the loss of E3 ligase activity of TRAF4 (C18A mutation) strikingly abrogated Akt ubiquitination and decreased Akt phosphorylation compared with wild-type (WT) TRAF4 (Fig. 4D). These changes, including reduced Akt ubiquitination and phosphorylation, were also observed in TRAF4-knockout CAL27R cells (Fig. 4E). Moreover, Flag-TRAF4 with HA-Akt-WT or K8/K14R mutant were co-transfected in CAL27R cells, and it was found that K8/K14R mutation compromised TRAF4-induced Akt ubiquitination and phosphorylation, indicating that TRAF4-induced Akt ubiquitination occurred on K8/K14 Lys residues (Fig. 4F). Depleting TRAF4 decreased Akt phosphorylation and GSK3β phosphorylation at Ser9, resulting in activation of GSK3β kinase, which contributed to upregulating MCL-1 phosphorylation and promoted its degradation in CAL27R and SCC25R cells exposed to IR for 20 min (Fig. 4G), indicating that TRAF4 is required for IR-induced Akt signaling activation. The present study further assessed the changes of these molecules in TRAF4-deficient CAL27R cells subjected to IR treatment for different times, and the IB data showed that blocking TRAF4 led to the downregulation of the Akt/MCL-1 signaling pathway in a time-dependent manner (Fig. 4H). GSK3β activation promoted protein MCL-1 phosphorylation at Ser159 and ubiquitination [29]. To examine whether GSK3β was essential for IR-induced MCL-1 reduction, GSK3β stable knockdown cell lines were constructed using shRNA in CAL27R and SCC25R cells. The results showed that depletion of GSK3β attenuated IR-induced MCL-1 S159 phosphorylation and restored MCL-1 protein levels (Fig. 4I). Consistent with this observation, suppression of GSK3β activity by the small molecular inhibitor SB216763 compromised MCL-1 reduction in TRAF4-null cells even in the presence of IR (Fig. 4J). Altogether, these findings suggest that the E3 ligase TRAF4 is required for IR-induced Akt activation, and knockout of TRAF4 promotes IR-induced MCL-1 reduction.

### MCL-1 phosphorylation disrupts the interaction between JOSD1 and MCL-1 in TRAF4- deficient OSCC cells

**Fig. 5:**
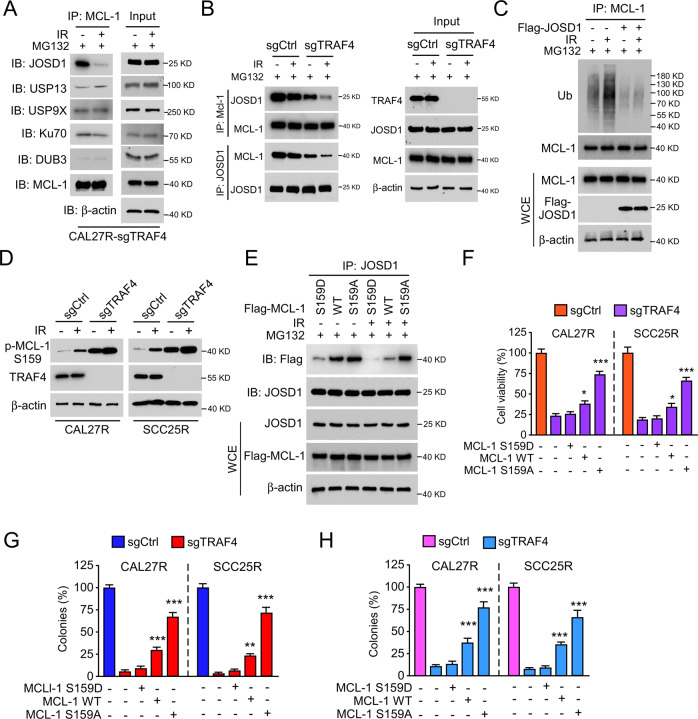
Irradiation disrupts the interaction between JOSD1 and MCL-1. **A** TRAF4 knockout CAL27R cells were treated with/without IR (2 Gy) for 20 min, and WCE was subjected to co-immunoprecipitation (Co-IP) analysis. **B** Immunoblotting for IP-mediated MCL-1 and JOSD1 expression in TRAF4 knockout CAL27R cells treated with/without IR (2 Gy) for 20 min. **C** CAL27R cells were transfected with Flag-JOSD1 for 48 h, followed by IR (2 Gy) treated for 20 min. IP assay was performed to detect MCL-1 ubiquitination. **D** Immunoblotting for p-MCL-1 S159 expression in TRAF4 knockout CAL27R and SCC25R cells treated with/without IR (2 Gy) for 72 h. **E** CAL27R cells were transfected with Flag-MCL1 WT, Flag-MCL1 S159D and Flag-MCL1 S159A for 48 h, followed by IR (2 Gy) treated for 20 min. WCE was subjected to co-immunoprecipitation (Co-IP) analysis. **F**–**H** Transfected with Flag-MCL1 S159A mutant restored proliferation of TRAF4 knockout OSCC cells. TRAF4 knockout CAL27R and SCC25R cells were transfected with Flag-MCL1 WT, Flag-MCL1 S159D and Flag-MCL1 S159A for 48 h. MTS assay was used to determine the cell viability (**F**), plate colony formation assay to analyze the colony formation (**G**) and soft agar assay to assess the anchorage-independent cell growth (**H**). All data are means ± s.e.m. **p < 0.01, ***p < 0.001, a significant difference between groups as indicated.

It has been previously reported that overexpression of the deubiquitinases, such as JOSD1, USP13, USP9X, Ku70 and DUB3, protect MCL-1 from proteasome degradation [30]. Co-IP assay was carried out to detect whether certain deubiquitinases were involved in MCL-1 reduction in TRAF4-deficient OSCC cells. As shown in Fig. 5A, the interaction between JOSD1 and MCL-1 was strikingly disrupted in TRAF4-deficient cells, while other deubiquitinases were not affected. Likewise, compared with CAL27R-sgCtrl cells, the depletion of TRAF4 destroyed the interaction between JOSD1 and MCL-1, which was further enhanced in the presence of radiation (Fig. 5B). Moreover, the ubiquitination assay showed that ectopic overexpression of JOSD1 remarkably abolished the IR-induced ubiquitination of MCL-1 in CAL27R cells (Fig. 5C). Importantly, cell viability (Fig. S1A), plate colony formation (Fig. S1B) and anchorage-independent colony formation (Fig. S1C) were significantly restored after transfection of Flag-JOSD1 in TRAF4-null CAL27R cells. Since MCL-1 ubiquitination is dependent on GSK3β-mediated MCL-1 S159 phosphorylation [31] and TRAF4 knockout increased the expression of MCL-1 S159 phosphorylation (Fig. 5D), it was speculated that the phosphorylation site Ser159 of MCL-1 may be the critical point for IR-induced MCL-1 reduction. Flag-MCL1 WT, Flag-MCL1 S159D mutant and Flag-MCL1 S159A mutant were ectopically expressed in OSCC cells to evaluate this hypothesis. MCL1 WT and S159D mutant (which is a phosphomimetic mutant in the GSK3β phosphorylation site), but not MCL1 S159A mutant (which abolished phosphorylation by GSK-3), substantially decreased the interaction between JOSD1 and MCL-1 in the presence of IR in CAL27R cells (Fig. 5E). Moreover, the MCL1 S159A mutant significantly recovered cell viability (Fig. 5F) and colony forming potential (Fig. 5G, H) in TRAF4-null CAL27R and SCC25R cells, whereas the MCL1 S159D mutant did not. These results suggest that the phosphorylation of MCL-1 S159 disrupts the interaction between JOSD1 and MCL-1, and eventually induces MCL-1 ubiquitination and degradation in TRAF4-knockout OSCC cells.

### TRAF4 knockout overcomes radioresistance in vivo

**Fig. 6:**
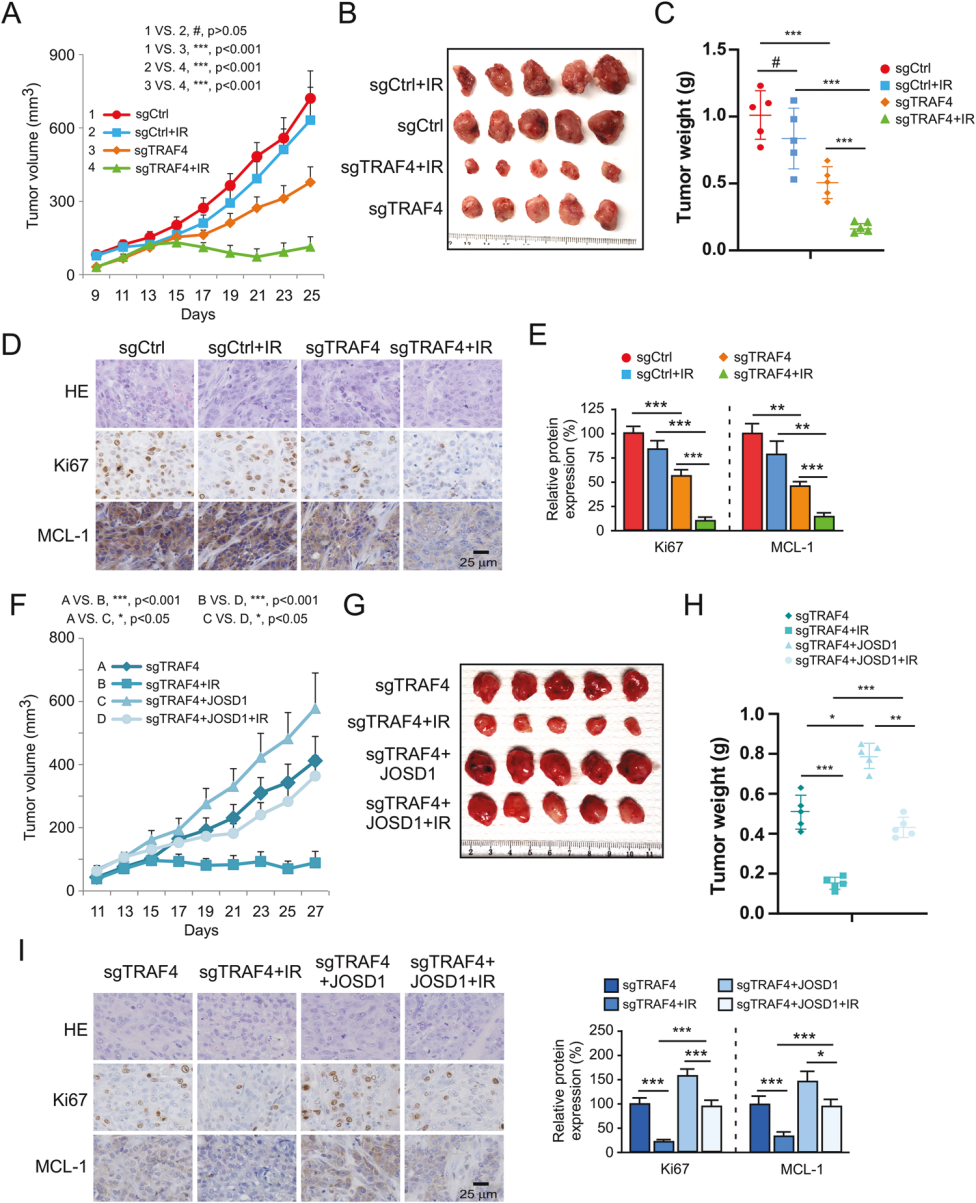
TRAF4 knockout overcomes radioresistance in vivo. **A**–**E** TRAF4 knockout sensitizes OSCC cells to radiotherapy. Xenograft tumors derived from TRAF4 knockout CAL27R cells were treated with/without IR (2 Gy) for 25 days; *n* = 5 mice per group. Tumor growth curves (**A**), tumor mass images (**B**), and tumor weight (**C**) were recorded. Tumor sections were subjected to IHC staining of Ki67 and MCL-1 (**D**), and qualification analysis of Ki67 and MCL-1 protein expression (**E**). Scale bar, 25 μm. **F**–**I** JOSD1 introduction into TRAF4 knockout CAL27R cells rescues tumorigenesis under IR treatment. Xenograft tumors derived from TRAF4 knockout and ectopic expression JOSD1 CAL27R cells were treated with/without IR (2 Gy) for 27 days; *n* = 5 mice per group. Tumor growth curves (**F**), tumor mass images (**G**), and tumor weight (**H**) were recorded. Tumor sections were subjected to IHC staining of Ki67 and MCL-1 (I, left), and qualification analysis of Ki67 and MCL-1 protein expression (I, right). Scale bar, 25 μm. All data are means ± s.e.m. *p < 0.05, **p < 0.01, ***p < 0.001, a significant difference between groups as indicated.

The results of in vitro experiments illustrated that blocking TRAF4 significantly re-sensitized OSCC cells to radiotherapy. Next, xenograft tumor models were generated using CAL27R cells expressing sgTRAF4 or sgCtrl. Compared with those of the Ctrl group, tumor growth (Fig. 6A), tumor mass (Fig. 6B), tumor weight (Fig. 6C) and tumor cell proliferation (Fig. 6D, E) were significantly decreased in the xenograft tumors derived from TRAF4 knockout. Notably, this inhibitory effect was further enhanced with combined IR treatment. IHC staining suggested that silencing of TRAF4 synergized with IR to reduce the protein level of MCL-1 (Fig. 6D, E). Next, the impact of MCL-1 ubiquitination on radiotherapeutic function was investigated. In the TRAF4-knockout xenograft tumors, the introduction of JOSD1 markedly attenuated the antitumor effects of blocking TRAF4 even in the presence of IR, as demonstrated by the tumor growth rate (Fig. 6F), tumor mass (Fig. 6G) and tumor weight (Fig. 6H), which significantly increased. Consistently, Ki-67-positive cells population and MCL-1 protein levels were recovered upon introduction of JOSD1 (Fig. 6I). These results indicate that depletion of TRAF4 sensitizes OSCC cells to radiotherapy, which depends on disrupting the interaction of JOSD1 with MCL-1 to promote MCL-1 ubiquitination and degradation.

### TRAF4 is highly expressed and positively correlates with MCL-1 in samples from patients with OSCC

**Fig. 7:**
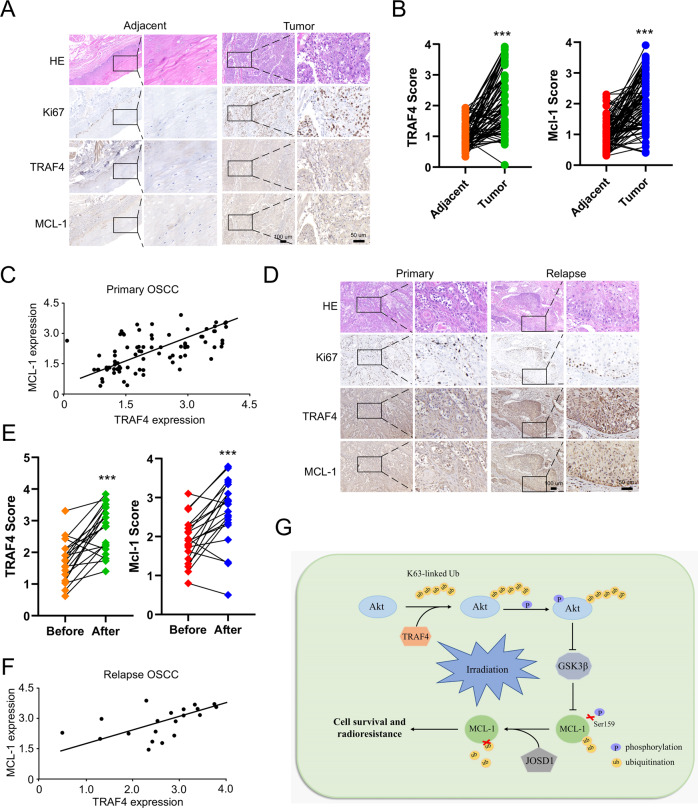
TRAF4 is highly expressed and positively correlates with MCL-1 in OSCC patient samples. **A** IHC staining was performed to determine Ki67, TRAF4 and MCL-1 from 81 cases of primary tumors with matched adjacent tissues. Scale bar, 50 μm. **B** IHC scores for TRAF4 and MCL-1 in 81 cases paired tumors and adjacent tissues. **C** MCL-1 expression is positively correlated with TRAF4 as scatterplot analysis. (r = 0.5147, *p* < 0.001). **D** IHC staining was performed to determine Ki67, TRAF4 and MCL-1 in 20 cases paired primary and relapsed tumor tissues. Scale bar, 50 μm. **E** IHC scores for TRAF4 and MCL-1 in 20 cases paired primary and relapsed tissues. **F** MCL-1 expression is positively correlated with TRAF4 in relapsed tumor tissues as scatterplot analysis. (r = 0.4635, *p* < 0.05) **G** The schematic of TRAF4-mediated regulation of MCL-1 expression and radioresistance. All data are means ± s.e.m. ***p < 0.001, a significant difference between groups as indicated.

To demonstrate the clinical relevance of the present findings, the TRAF4 and MCL-1 protein levels were examined in 81 primary OSCC tissues. The expression of TRAF4 and MCL-1 were significantly upregulated in tumor tissues compared with that in paired adjacent non-tumor tissues (Fig. 7A, B; Table 1). There was a positive correlation between TRAF4 and MCL-1 expression (Fig. 7C). The correlation between TRAF4 and MCL-1, and the sensitivity to radiotherapy were further confirmed in 20 cases of paired OSCC specimens before and at relapsed after radiotherapy. The IHC data showed that the Ki67-positive cells population was not changed between the primary and relapsed tumor tissues (Fig. 7D). Interestingly, TRAF4 and MCL-1 levels exhibited significantly higher levels in relapsed tumor tissues than in primary tumor tissues (Fig. 7D, E; Table 2), and the positive correlation between TRAF4 and MCL-1 was also observed in the relapsed tumors (Fig. 7F). These findings suggest a positive correlation between TRAF4 and MCL-1 proteins, which may play a crucial role in tumorigenesis and radioresistance in OSCC.

**Table 1:** Quantification of TRAF4 and MCL-1 protein levels in normal and OSCC tissues.

Characteristic	Tumor (*n* = 81)	Adjacent (*n* = 81)	*P* value
*TRAF4*			
High	36	0	*p* < 0.001
Low	45	81	
*MCL-1*			
High	43	4	*p* < 0.001
Low	38	77

**Table 2:** Quantification of TRAF4 and MCL-1 protein levels in primary (before) and relapsed (after) OSCC tissues.

Characteristic	Before (*n* = 20)	After (*n* = 20)	*P* value
*TRAF4*			
High	6	16	*p* < 0.05
Low	14	4	
*MCL-1*			
High	8	16	*p* < 0.01
Low	12	4	

### Inhibiting MCL-1 overcomes radioresistance in OSCC cells

**Fig. 8:**
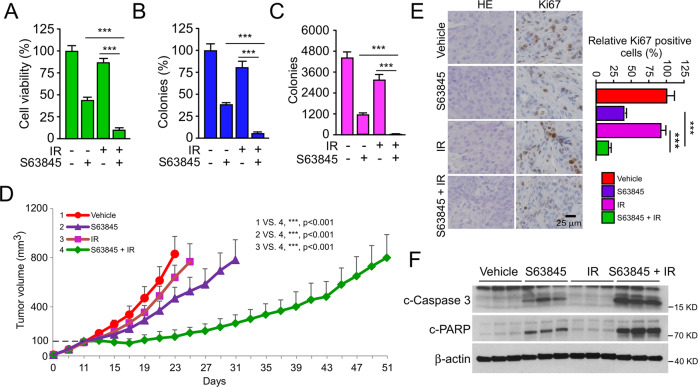
Inhibiting MCL-1 overcomes radioresistance of OSCC in vivo. **A**–**C** CAL27R cells were treated with S63845 inhibitor (2 µM), IR (2 Gy) or a S63845/IR combination for 48 h. MTS assay was used to determine the cell viability (**A**), plate colony formation assay to analyze the colony formation (**B**) and soft agar assay to assess the anchorage-independent cell growth (**C**). **D**, **E** Xenograft tumors derived from CAL27R cells were treated with vehicle control (0.5% DMSO in Corn oil, 100 µL/every 2 days, i.p.), S63845 (5 mg/kg/ in 100 µL Corn oil every 2 days, i.p), IR (2 Gy/twice per week) or a S63845/IR combination when tumor volume reached 100 mm^3^. *n* = 5 mice per group. Tumor growth curves (**D**) were recorded and tumor sections were subjected to IHC staining of Ki67 (**E**). Scale bar, 25 μm. **F** Immunoblotting for c-caspase 3 and c-PARP expression in CAL27R cells treated with vehicle control, S63845, IR or a S63845/IR combination. All data are means ± s.e.m. ***p < 0.001.

To further verify the role of the TRAF4/MCL-1 signaling pathway in the radioresistance of OSCC, the specific small molecule inhibitor of MCL-1, S63845, was used in subsequent experiments in vitro and in vivo experiments. The results showed that the combination of S63845 and IR significantly reduced cell viability (Fig. 8A) and colony formation (Fig. 8B, C) in CAL27R cells compared with those in cells treated with S63845 or IR alone. Similarly, the same inhibitory effect was observed in SCC25R cells (Fig. S2A-C). Next, the CAL27R xenograft tumors were generated, and the animals were administrated with S63845, IR or a combination of both when the tumor volume reached 100 mm^3^. It was found that combining S63845 with IR substantially delayed the growth of tumors (Fig. 8D). Consistently, the IHC results revealed that the population of Ki67-positive cells was noticeably decreased in CAL27R cells treated with S63845 and IR (Fig. 8E). As expected, intrinsic apoptosis was activated following S63845 treatment, and was further enhanced in combination with IR. As the IB data showed, the expression of cleaved-caspase 3 and cleaved-PARP was increased obviously (Fig. 8F). Altogether, these results suggest that the dysregulation of MCL-1-mediated intrinsic apoptosis plays a critical role in the radioresistance of OSCC, and that targeting MCL-1 may be a promising therapeutic strategy to overcome radioresistance in OSCC.

## Discussion

Radiation resistance remains the leading cause of radiotherapy failure in patients with OSCC, resulting in post-IR recurrence and poor clinical outcomes [32, 33]. However, only a few biomarkers are currently available to explain the potential mechanism of radioresistance [34, 35]. For example, nuclear factor erythroid 2-related factor 2 (Nrf2) was recently shown to be upregulated in clinically relevant radioresistant OSCC cells, and to be involved in the development of IR resistance via enhancing Nrf2-dependent glycolysis and glutathione synthesis [34]. Chen et al. found that 60S ribosomal protein L36a (RPL36A) was closely related to poor prognosis in patients with OSCC treated with radiotherapy, and depletion of RPL36A sensitized OSCC cells to DNA damage and promoted G_2_/M cell cycle arrest to upregulate IR-induced apoptosis, which led to increased radiosensitivity in OSCC cells [23]. MicroRNA-125b was reported to be downregulated in OSCC cells, and exhibited an antitumor effect by reducing cell proliferation and enhancing radiosensitivity through the regulation of intercellular adhesion molecule-2 signaling [36]. Therefore, further studies on biomarkers and the molecular pathways that are involved in radiation resistance is of great importance to better understand radioresistance characteristics and to formulate effective treatment strategies to improve the overall survival of patients with OSCC undergoing radiotherapy. The present study first determined that TRAF4 was overexpressed in radioresistant OSCC cell lines and tissues. Depletion of TRAF4 significantly increased the sensitivity of OSCC cells to radiation in vitro and in vivo. These findings suggest a critical correlation between TRAF4 and radioresistance in OSCC, indicating that TRAF4 may be an attractive targeted biomarker to counteract radioresistance.

IR induces cell death by exerting its cytotoxic effects, such as activation of intrinsic apoptosis [37]. This is consistent with the fact that the inhibitor z-VAD-fmk compromised the IR-induced downregulation of cell viability in OSCC cells. Notably, this effect was also detected in TRAF4-knockout radioresistant OSCC cells, indicating that intrinsic apoptosis was responsible for blocking TRAF4 to recover the sensitivity of radioresistant OSCC cells to radiation. The antiapoptotic Bcl-2 family, including Bcl-2, Bcl-xL, Bcl-w, Bcl-2-A1, and MCL-1, plays an important role in regulating intrinsic apoptosis [38]. MCL-1, a crucial member of the Bcl-2 family, is frequently overexpressed in human tumors, and is a critical cause of chemoresistance and radioresistance [39–41]. The mRNA and protein expression of MCL-1 was found to be elevated in chemoresistant OSCC cells, and inhibition of MCL-1 using genetic or pharmacological methods induced cell death in OSCC cells resistant to either cisplatin, fluorouracil or docetaxel [42]. Radiation-induced stemness and radioresistance in nasopharyngeal carcinoma (NPC) largely depended on the upregulation of MCL-1 protein, which was attributed to intracellular reactive oxygen species and Akt activation following repeated ionizing radiation [43]. MCL-1 expression was also associated with deubiquitinase USP9X-mediated radioresistance in hematological tumors [44]. Notably, the current study revealed that MCL-1 was overexpressed in tumor tissues, particularly in relapsed tumor samples of patients with OSCC. There was a positive correlation between TRAF4 and MCL-1 in primary and relapsed tumor samples. Knockout of TRAF4 decreased MCL-1 expression and accelerated its degradation by upregulating IR-induced MCL-1 ubiquitination. These results suggested that TRAF4-mediated downregulation of the sensitivity of OSCC cells to radiotherapy was primarily attributable to modulating the expression of the candidate substrate MCL-1.

As an E3 ubiquitin ligase, TRAF4 can catalyze ubiquitination to degrade or activate its target substrates. A previous study showed that TRAF4 promoted the degradation of SMAD ubiquitination regulatory factor 2 (SMURF2) by increasing the polyubiquitination of SMURF2 to regulate pro-oncogenic TGF-β signaling and promote breast cancer metastasis [45]. TRAF4 was found to catalyze the ubiquitination of the DNA-damage checkpoint kinase 1 (CHK1) at the K132 site to induce its phosphorylation and activation, resulting in chemotherapy resistance in colorectal cancer [46]. Deregulation of Akt signaling exerts an important role in tumorigenesis via modulation of the proliferation, survival and metabolism of tumor cells. Akt is constitutively activated in response to various stimuli, such as growth factors, hormones, and stressors. Previous studies detailing Akt activation indicated that E3 ligases-mediated Akt ubiquitination was required for Akt irritant activation. Yu et al. found that EGF-induced Akt mitochondrial localization and activation in nasopharyngeal carcinoma (NPC) cells was dependent on S-phase kinase-associated protein 2 (Skp2)-mediated Akt K63-linked ubiquitination [22]. In addition, E3 ligase TRAF6-induced Akt Lys 63 chain ubiquitination was essential for Akt membrane recruitment and phosphorylation upon IGF-1 stimulation [47]. Detailed analysis of the underlying mechanism in the current study revealed that IR markedly enhanced the interaction between TRAF4 and Akt, and also promoted TRAF4-mediated Akt K63-linked ubiquitination to activate Akt signaling. Furthermore, it was observed that TRAF4 knockout markedly deregulated the phosphorylation of Akt and GSK3β S9, leading to increased GSK3β activity, which upregulated MCL-1 phosphorylation and promoted its degradation. The current study is in agreement with previous research that indicated that Akt/GSK3β signaling is required for MCL-1 S159 phosphorylation and degradation [48]. Certain deubiquitinases such as USP9X [49], JOSD1 [50], USP13 [51], DUB3 [52] and Ku70 [53] have been demonstrated to stabilize MCL-1. However, the molecular mechanisms by which these deubiquitinases stabilize MCL-1 are not fully understood. Our results indicated that depletion of TRAF4 disrupted the interaction between MCL-1 and JOSD1, particularly under IR treatment. Further molecular mechanistic studies showed for the first time that MCL-1 S159 phosphorylation was the main factor that interfered with JOSD1 interaction. Next, xenograft tumor models were generated to validate the results of the above in vitro observations. The results showed that TRAF4 knockout or suppression of MCL-1 with S63845 combined with radiotherapy could significantly inhibit tumor growth. In contrast, after the introduction of JOSD1, the sensitivity and efficacy of IR remarkably reduced. Thus, the current data suggested that TRAF4-mediated MCL-1 degradation was the ultimate reason for the increased radiosensitivity of OSCC cells. However, further research is required to explore whether other signaling pathways or biomolecules are involved in the TRAF4-regulated MCL-1 expression.

Collectively, the present study demonstrated that TRAF4 was highly expressed in radioresistant OSCC cell lines and clinical OSCC tissues. TRAF4 promoted Akt K63-linked ubiquitination and activation to suppress GSK3β activity and MCL-1 S159 phosphorylation, facilitating the interaction between MCL-1 and JOSD1, which ultimately promoted the stability and expression of MCL-1 to confer resistance to radiotherapy in OSCC cells (Fig. 7G). The present study provided insights into the functional role and molecular mechanism of TRAF4 in OSCC radioresistance, suggesting that TRAF4 may be a potential target to overcome radioresistance in OSCC.

## Supplementary information

supplemental information

Original Data File

Reproducibility checklist

## Data Availability

The datasets generated and/or analyzed during the current study are available from the corresponding author on reasonable request.
