# Correction to: Stabilization of MCL-1 by E3 ligase TRAF4 confers radioresistance

**DOI:** 10.1038/s41419-022-05533-x

**Published:** 2023-01-18

**Authors:** Ming Li, Feng Gao, Xiaoying Li, Yu Gan, Shuangze Han, Xinfang Yu, Haidan Liu, Wei Li

**Affiliations:** 1 School of Stomatology, Hunan University of Chinese Medicine, Changsha, Hunan 410208 People’s Republic of China; 2 Changsha Stomatological Hospital, Changsha, Hunan 410004 People’s Republic of China; 3 Cell Transplantation and Gene Therapy Institute, The Third Xiangya Hospital of Central South University, Changsha, Hunan 410013 People’s Republic of China; 4 Department of Radiology, The Third Xiangya Hospital of Central South University, Changsha, Hunan 410013 People’s Republic of China; 5 Department of Ultrasonography, The Third Xiangya Hospital of Central South University, Changsha, Hunan 410013 People’s Republic of China; 6 Department of Ultrasound, Union Hospital, Tongji Medical College, Huazhong University of Science and Technology, Wuhan, Hubei 430022 People’s Republic of China; 7 Department of Medicine, Baylor College of Medicine, Houston, TX 77030 USA; 8 Department of Cardiovascular Surgery, The Second Xiangya Hospital of Central South University, Changsha, Hunan 410011 People’s Republic of China; 9 Clinical Center for Gene Diagnosis and Therapy, The Second Xiangya Hospital of Central South University, Changsha, Hunan 410011 People’s Republic of China

**Keywords:** Oral cancer, Tumour biomarkers

Correction to: *Cell Death and Disease* 10.1038/s41419-022-05500-6, published online 19 December 2022

The original version of this article contained an error in the affiliations. Dr Ming Li is also affiliated with the Department of Radiology, The Third Xiangya Hospital of Central South University, Changsha, Hunan 410013, People’s Republic of China as well as with the Cell Transplantation and Gene Therapy Institute, The Third Xiangya Hospital of Central South University, Changsha, Hunan 410013, People’s Republic of China. The original article has been corrected.

