# Supplementary material for: Stabilization of MCL-1 by E3 ligase TRAF4 confers radioresistance
Source: Cell Death Dis. 2022 Dec 19;13(12):1053. doi: 10.1038/s41419-022-05500-6 (PMC9763423; doi:10.1038/s41419-022-05500-6)
Supplement: Supplementary file 1 — supplemental information [file 41419_2022_5500_MOESM1_ESM.pdf]

# Figure S1

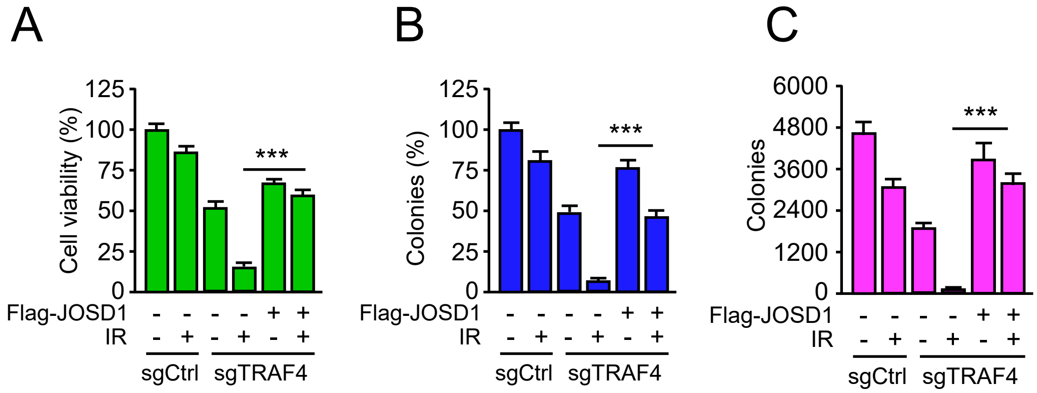
A
B
C
Cell viability (%)
Colonies (%)
Colonies
125
100
75
50
25
0
6000
4800
3600
2400
1200
***
Flag-JOSD1 - - - - + +
IR - + - + - +
sgCtrl
sgTRAF4

# Figure S2

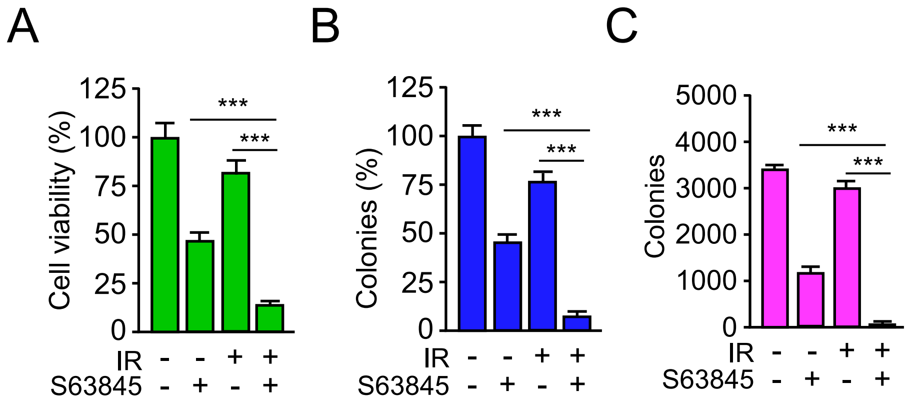

A
B
C
Cell viability (%)
Colonies (%)
Colonies
125
100
75
50
25
0
5000
4000
3000
2000
1000
***
IR - - + +
S63845 - + - +

**Table 1: Quantification of TRAF4 and MCL-1 protein levels in normal and OSCC tissues**

| Characteristic | Tumor (n=81) | Adjacent (n=81) | *P* value |
|---|---|---|---|
| TRAF4 | | | |
| High | 36 | 0 | $p$ <0.001 |
| Low | 45 | 81 | |
| MCL-1 | | | |
| High | 43 | 4 | $p$ <0.001 |
| Low | 38 | 77 | |

**Table 2: Quantification of TRAF4 and MCL-1 protein levels in primary (before) and relapsed (after) OSCC tissues**

| Characteristic | Before (n=20) | After (n=20) | *P* value |
|---|---|---|---|
| TRAF4 | | | |
| High | 6 | 16 | $p$ <0.05 |
| Low | 14 | 4 | |
| MCL-1 | | | |
| High | 8 | 16 | $p$ <0.01 |
| Low | 12 | 4 | |

**Supplementary Figure Legends**

Supplementary Figure 1. A-C. Ectopic expression JOSD1 rescues tumorigenesis in TRAF4 knockout CAL27R cells under IR treatment. CAL27R cells were transfected with Flag-JOSD1 for 48 h, followed by IR (2 Gy) treated for 20 min. MTS assay was used to determine the cell viability (A), plate colony formation assay to analyze the colony formation (B) and soft agar assay to assess the anchorage-independent cell growth (C). ***$p$ <0.001.

Supplementary Figure 2. A-C. SCC25R cells were treated with S63845 inhibitor (2 µM), IR (2 Gy) or a S63845/IR combination for 48 h. MTS assay was used to determine the cell viability (A), plate colony formation assay to analyze the colony formation (B) and soft agar assay to assess the anchorage-independent cell growth (C). ***$p$ <0.001.
