# Supplementary material for: Stabilization of MCL-1 by E3 ligase TRAF4 confers radioresistance
Source: Cell Death Dis. 2022 Dec 19;13(12):1053. doi: 10.1038/s41419-022-05500-6 (PMC9763423; doi:10.1038/s41419-022-05500-6)

Figure S3 a, Full gel for Figure 1

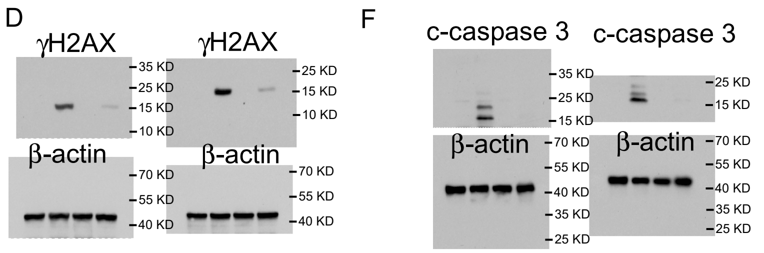

Figure S3 b, Full gel for Figure 2

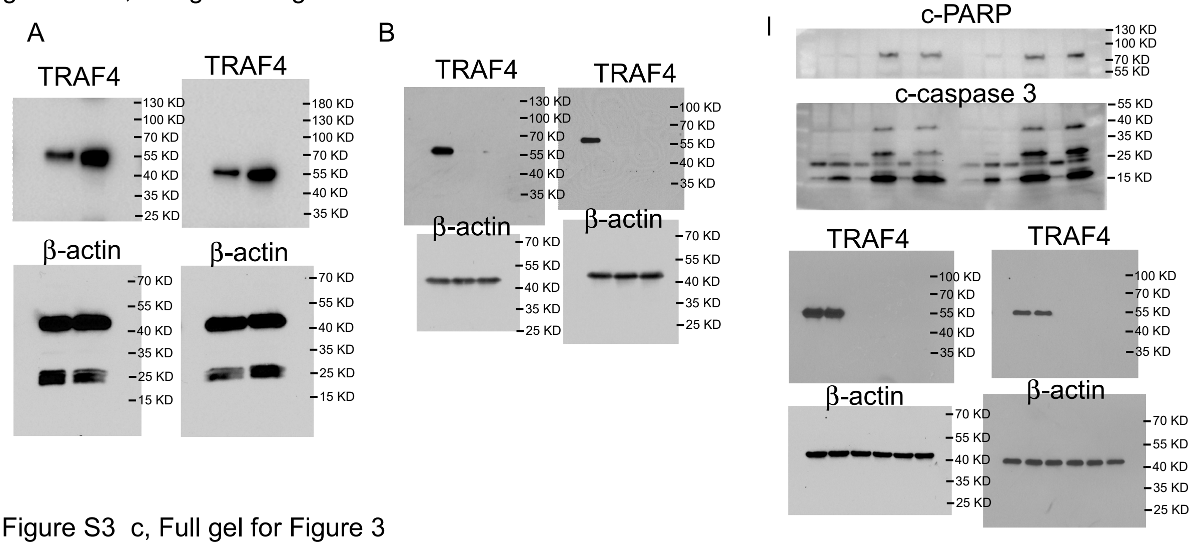

Figure S3 c, Full gel for Figure 3

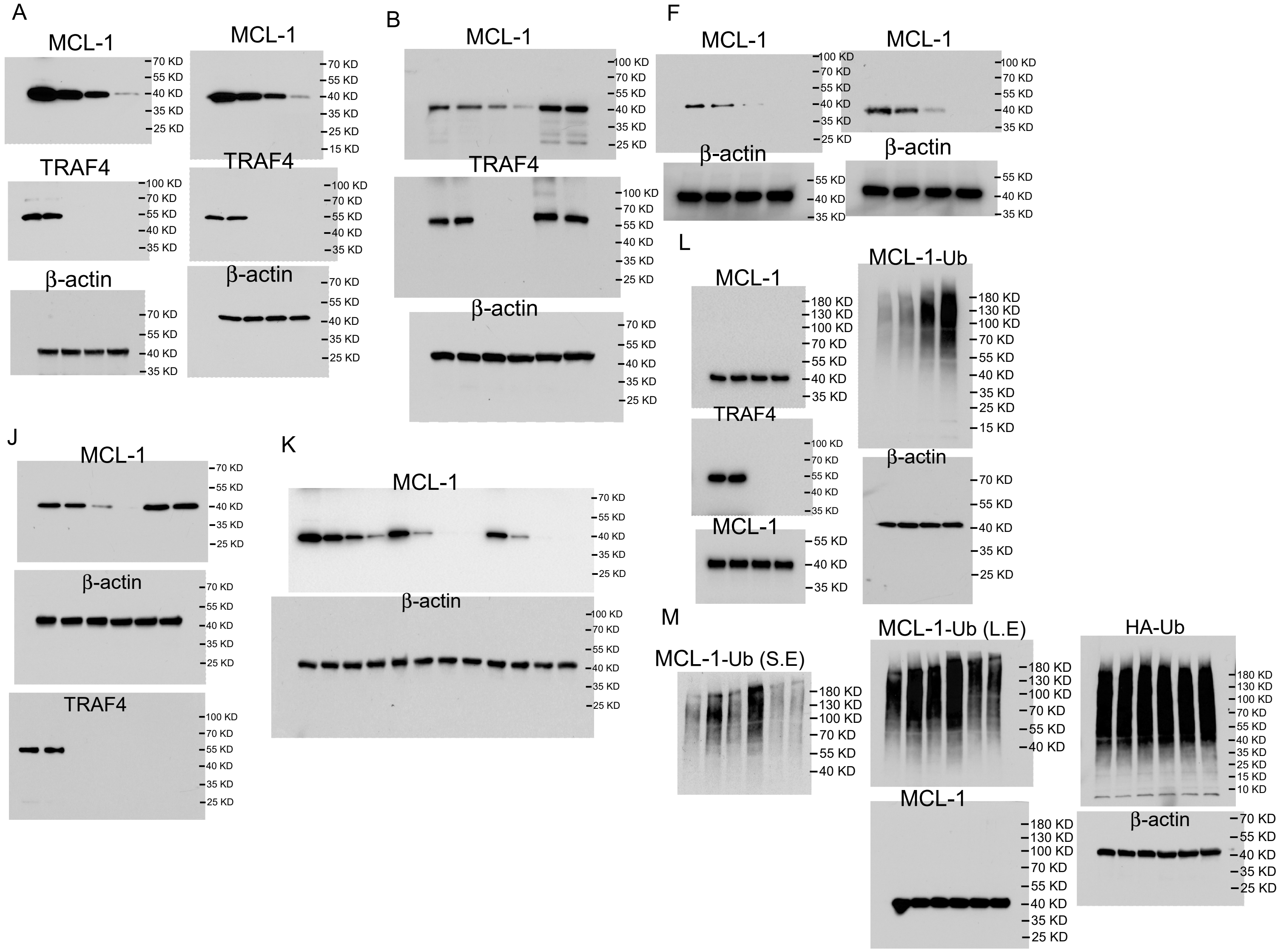

Figure S3 d, Full gel for Figure 4

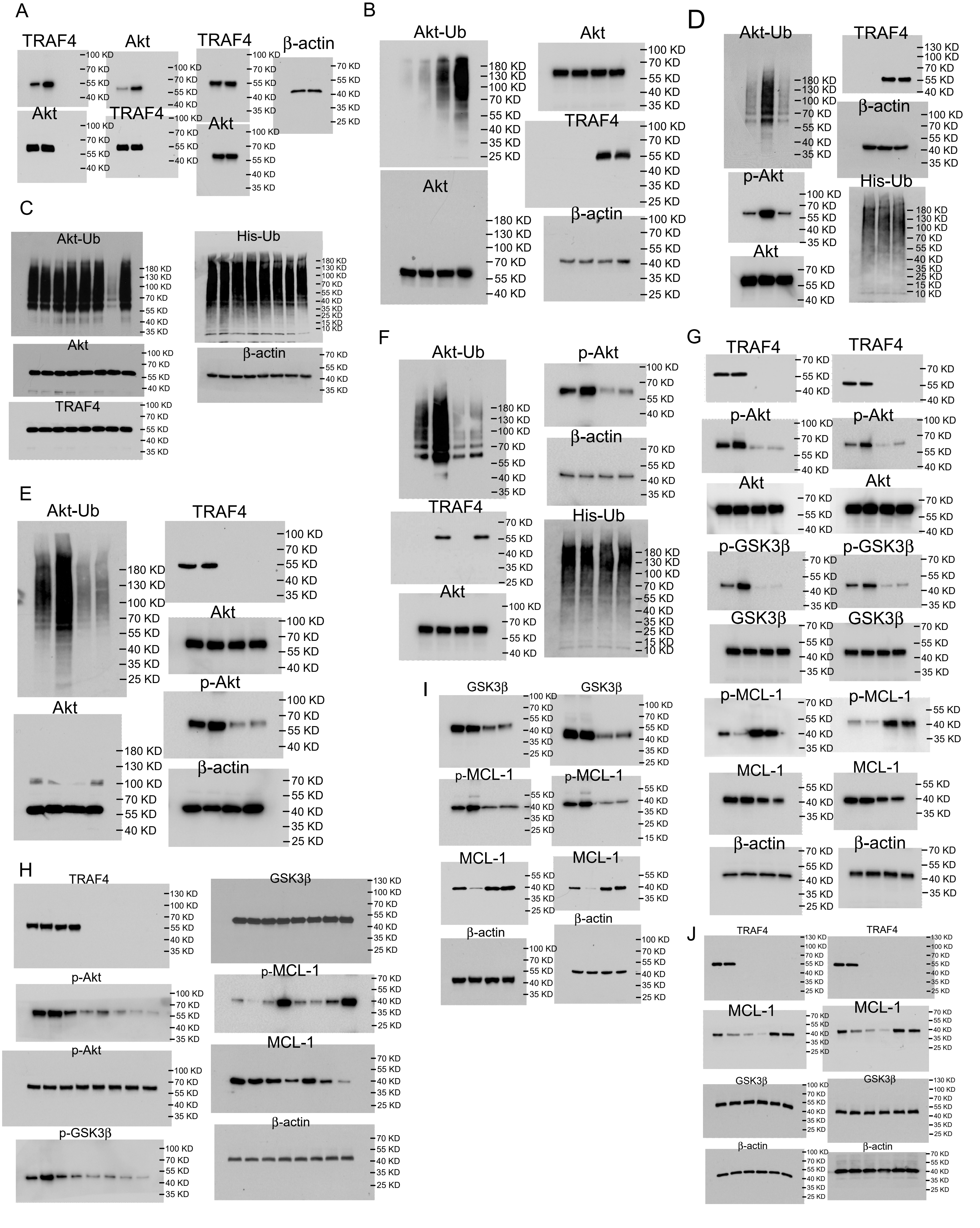

Figure S3 e, Full gel for Figure 5

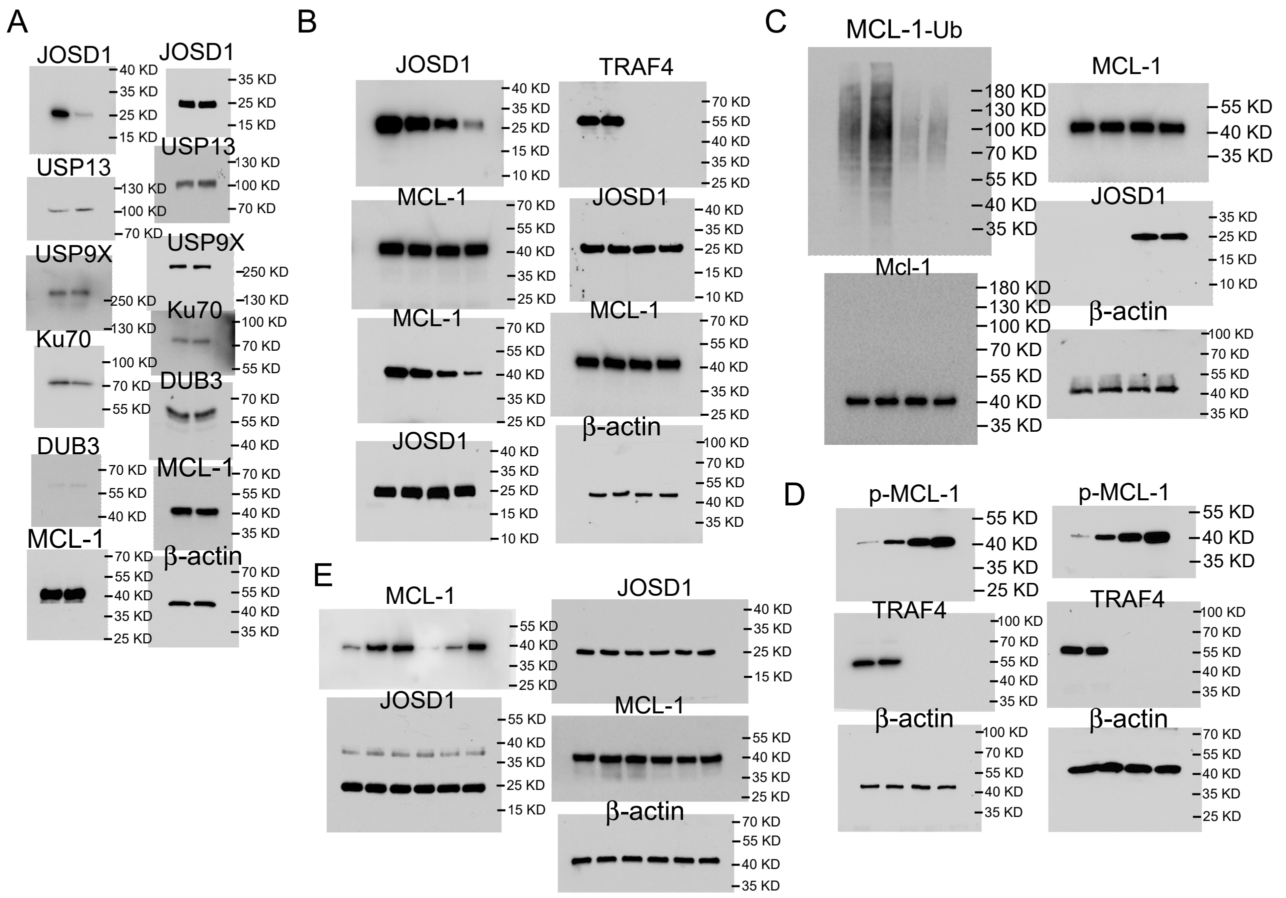

Figure S3 f, Full gel for Figure 8

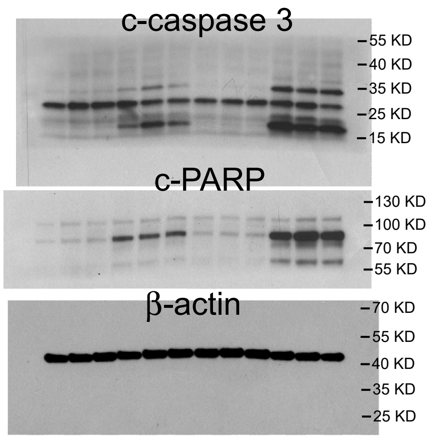

Supplement: Supplementary file 2 — Original Data File [file 41419_2022_5500_MOESM2_ESM.pdf]
